# Risk Factors for Benign Anastomotic Stenosis After Esophagectomy for Cancer

**DOI:** 10.1245/s10434-025-17401-x

**Published:** 2025-05-06

**Authors:** Dillen C. van der Aa, Jelle Boonstra, Wietse J. Eshuis, Freek Daams, Roos E. Pouw, Suzanne S. Gisbertz, Mark I. van Berge Henegouwen

**Affiliations:** 1 Department of Surgery, Amsterdam UMC, Location University of Amsterdam, Amsterdam, The Netherlands; 2 Cancer Center Amsterdam, Cancer Treatment and Quality of Life, Amsterdam, The Netherlands; 3 Amsterdam Gastroenterology Endocrinology Metabolism, Amsterdam UMC location, University of Amsterdam, Amsterdam, The Netherlands; 4 Department of Gastroenterology and Hepatology, Amsterdam UMC location Vrije Universiteit Amsterdam, Amsterdam, The Netherlands

**Keywords:** Esophagectomy, Stenosis, Stricture, Benign

## Abstract

**Background:**

Benign stenosis frequently occurs after esophagectomy, causing dysphagia, eating problems, and diminished quality of life. This study aimed to identify risk factors for benign anastomotic stenosis after esophagectomy for cancer.

**Methods:**

This retrospective cohort study analyzed patients who underwent esophagectomy at Amsterdam UMC from 2012 until 2022. Intrathoracic and cervical anastomoses were examined separately. Benign anastomotic stenosis was defined as stenosis at the anastomosis causing dysphagia (Ogilvie score ≥2) and requiring at least one endoscopic dilation. Predictive factors were identified using logistic regression.

**Results:**

The study enrolled 902 patients: 605 with intrathoracic and 297 with cervical anastomosis. Of these cases, 91.1 % were a minimally invasive esophagectomy. Stenosis occurred in 18.4 % of the intrathoracic cases and 49.8 % of the cervical cases (*p* < 0.001). The patients required medians of 4 and 7 dilations, respectively (*p* = 0.001). The median time to stenosis was 99 days for the intrathor days for the cervical anastomoses (*p* = 0.001). Intrathoracic stenosis was independently associated with anastomotic leakage (odds ratio [OR], 2.034; 95 % confidence interval [CI], 1.116–3.708). For the patients without leakage, a 2 mm versus a 25 mm circular stapler reduced stenosis risk (OR, 0.486; 95 % CI, 0.294–0.803), whereas use of immunosuppressants (OR, 3.492; 95 % CI, 1.186–10.279]) and chronic pulmonary disease (OR, 2.717; 95 % CI, 1.293–5.707) increased it. For cervical anastomoses, hand-sewn end-to-side anastomosis was protective (OR, 0.454; 95 % CI, 0.234–0.879).

**Conclusions:**

The key risk factors for intrathoracic benign anastomotic stenosis are anastomotic leakage, smaller circular stapler size, use of immunosuppressants, and chronic pulmonary disease. For cervical anastomoses, the hand-sewn end-to side technique is protective compared with the end-to-end technique, whereas use of immunosuppressants and chronic pulmonary disease increases the risk.

**Supplementary Information:**

The online version contains supplementary material available at 10.1245/s10434-025-17401-x.

Esophageal cancer currently is the 11th most prevalent malignancy globally and the 7th most common cause of cancer mortality.^1^ The incidence in Europe is increasing. The Netherlands has the second highest incidence in Europe, with predominantly adenocarcinomas.^2,3^

Curative treatment of esophageal cancer consists of neoadjuvant or perioperative chemo (radio)therapy, followed by esophagectomy with lymphadenectomy. Esophagectomy is a complex surgical procedure with a morbidity rate of 65 %, even in high-volume, experienced centers, and a mortality rate ranging from 2 to 4%.^4^ Morbidity includes anastomotic leakage and respiratory complications, frequently leading to multiple reinterventions and intensive care unit (ICU) admission.^5^

During the past decades, advances in perioperative care have led to decreased morbidity and mortality rates, accompanied by an improved overall survival rate of up to 50 %.^2,3,6–8^ Therefore long-term outcomes have become increasingly important. However, despite these advancements, a prominent contributor to enduring morbidity after esophagectomy with gastric conduit reconstruction is the occurrence of benign anastomotic strictures, affecting 14 to 42% of patients.^9,10^ Recognized for their significant burden on patients’ quality of life, stenosis are characterized by a decline in nutritional status, difficulties with maintaining weight, difficulty with swallowing, an elevated risk of aspiration, and often, multiple interventions.^11,12^ Quality of life after an esophagectomy may be improved by the identification and mitigation of risk factors for anastomotic stenosis.

The literature exhibits contradicting results regarding risk factors for benign anastomotic strictures, particularly in this era of minimally invasive surgery, in which evidence is generally still lacking. Although anastomotic leakage is commonly associated with the strictures, its direct causality remains unproven.^10–13^ Challenges persist due to inconsistent reporting and terminology, making it difficult to assess changes despite declining anastomotic leakage rates. Surgeons often base their technique and choice of instruments, such as staplers, on personal preference and experience.^14^. Additionally, conflicting evidence exists regarding stapler techniques versus manual suturing.^15–18^

This study aimed to identify modifiable risk factors and high-risk patients for benign anastomotic stenosis after oncologic esophagectomy specific to location of the anastomosis.

## Patients and Methods

### Study Design

This single-center retrospective cohort study was conducted using a prospectively maintained database that included all patients with esophageal and gastroesophageal junction cancer who underwent esophagectomy from 2012 to the end of 2022 at the Amsterdam UMC, a tertiary referral center for esophageal cancer treatment. Data were collected for our institutional database by a retrospective search of the individual patient files for anastomotic stenosis in EPIC and Castor EDC.

Written informed consent was obtained from all patients to use their data. The study adhered to the Strengthening the Reporting of Observational Studies in Epidemiology (STROBE) guidelines to ensure accurate reporting of the research methods and findings.

### Patient Population

**Fig. 1:**
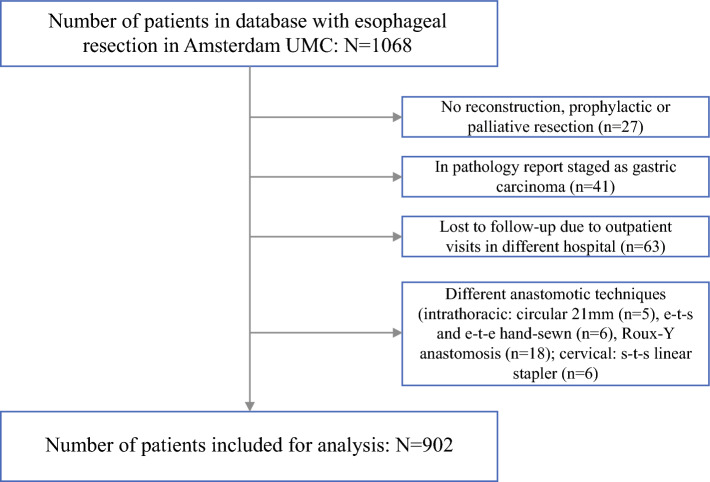
Flowchart of patient selection

The patient population included all patients older than 18 years with esophageal carcinoma (stages T1-4a, N1-3, M0) who underwent esophagectomy and reconstruction during the period 2012 to 2022 (1068 patients). The exclusion criteria ruled out absence of informed consent and follow-up evaluation received at another hospital. After the exclusion criteria were applied, 902 patients remained (Fig. 1).

### Surgery

The operative techniques (transthoracic esophagectomy, transhiatal esophagectomy, colonic interposition, and intrathoracic and cervical anastomosis) in the Amsterdam UMC have been described in detail previously^13^ and in the supplementary material. The procedure of choice is an Ivor Lewis procedure. The choice for a cervical anastomosis was determined at the discretion of the multidisciplinary team but was generally used for proximal esophageal tumors, proximal radiation fields, and high located (PET-positive) lymph nodes in the mediastinum.

### Follow-up Evaluation

Follow-up evaluation included regular visits to the outpatient clinic as per the national standards. These appointments occurred every 3 months during the first year and every 6 months from year 2 to year 4. One final visit occurred 5 years after the surgery. On indication, investigations were performed (e.g., a computed tomography [CT] scan, a positron emission tomography [PET]-CT scan, or an endoscopy.

### Anastomotic Stenosis

**Fig. 2:**
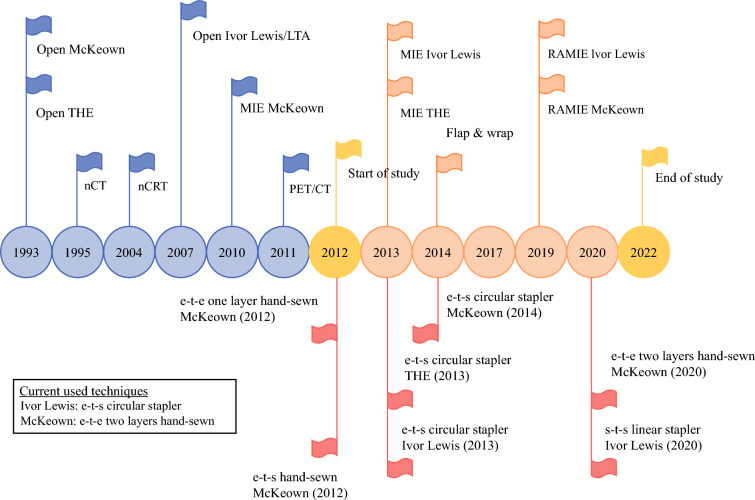
Changes in esophageal cancer treatment and anastomotic techniques. e-t-e, end-to-end; e-t-s, end-to-side; s-t-s, side-to-side; LTA, left thoracoabdominal; MIE, minimally invasive esophagectomy; nCRT, neoadjuvant chemoradiotherapy; nCT, neoadjuvant chemotherapy; PET/CT, positron emission tomography/computer tomography; RAMIE, robot-assisted minimally invasive esophagectomy; THE, transhiatal esophagectomy

Benign anastomotic stenosis was defined as the occurrence of postoperative dysphagia (Ogilvie ≥2 score denoting ability to swallow only semisolid foods^19^) requiring at least one endoscopic procedure to dilate the anastomosis in the absence of local anastomotic recurrent disease following United Kingdom guidelines.^20^ Time to anastomotic stenosis was determined as the time from the date of surgery until the date of the first endoscopic dilation (Fig.2).

### Endoscopic Dilation

Endoscopic procedures were performed with the patient under conscious or deep sedation. Endoscopic dilations were performed using bougies or controlled radial expansion balloons at the discretion of the endoscopist. Dilation followed the “rule of three,” in which the stricture was dilated by no more than 3 mm per session when resistance was encountered. The target diameter was at least 16 or 18 mm in case of recurrent strictures. In case of refractory or recurrent strictures, patients either received incision therapy or were taught self-dilation.

### Outcome Measures

The primary outcome was the incidence of benign anastomotic stenosis specified as to location and technique of anastomosis. The secondary outcomes were positive or negative predictors for benign anastomotic stenosis, postoperative complications, anastomotic leakage percentage, and number of dilations.

### Statistical Analysis

Categorical data are presented as number of cases and percentages, whereas continuous variables are reported as means with standard deviations or as medians with interquartile ranges (IQRs), depending on their distribution. Categorical variables are presented as frequencies and percentages.

The significance of the categorical descriptive statistics was calculated using chi-square for normally distributed categorical variables and Fisher’s exact test for non-normally distributed categorical variables. The significance for continuous descriptive statistics was calculated using one-way analysis of variance (ANOVA) for normally distributed variables and the Kruskal-Wallis test for non-normally distributed variables.

Data were analyzed using IBM SPSS statistics, version 28.0 (IBM, Chicago, IL, USA). Characteristics were calculated using univariable binary logistic regression. Factors with a *p* value lower than 0.200 were included in a multivariable binary logistic regression model, after which backward selection was used. A *p* value lower than 0.05 was considered statistically significant (Tables 1and 2).

**Table 1:** Baseline characteristics

	Intrathoracic	Cervical
Factor	(*n* = 605) *n* (%)	(*n* = 297) *n* (%)
Sex		
Male	495 (81.8)	198 (66.7)
Female	110 (18.2)	99 (33.3)
Mean age (years)	64.9 ± 15.5	64.0 ± 9.7
Median BMI: kg/m^2^ (range)	25.5 (14–47.6)	24.6 (15.4–43.7)
Comorbidities		
Hypertension	90 (14.9)	18 (6.1)
Atrial fibrillation	21 (3.5)	3 (1.0)
Chronic pulmonary disease	44 (7.3)	24 (8.1)
Diabetes mellitus	75 (12.4)	34 (11.4)
Liver disease	5 (0.8)	1 (0.3)
Myocardial infarction	25 (4.1)	2 (0.7)
Peripheral arterial disease	24 (4.0)	2 (0.7)
Renal disease	8 (1.3)	2 (0.7)
Use of immunosuppressants	19 (3.1)	5 (1.7)
Tumor location		
Cervical	0 (0.0)	2 (0.7)
Upper thoracic	0 (0.0)	9 (3.1)
Mid thoracic	40 (96.6)	75 (26.0)
Lower thoracic	441 (73.3)	156 (54.2)
Esophageal-cardia junction	119 (19.8)	45 (15.6)
Neoadjuvant treatment		
Neoadjuvant chemoradiation	515 (85.1)	245 (82.5)
Neoadjuvant chemotherapy	43 (7.1)	6 (2.0)
Neoadjuvant radiotherapy	1 (0.2)	0 (0.0)
No neoadjuvant treatment	46 (7.6)	46 (15.5)
Histologic type		
Adenocarcinoma	523 (86.4)	171 (57.6)
Squamous cell carcinoma	68 (11.2)	104 (35.0)
Other	14 (2.3)	22 (7.4)
Approach		
MIE or hybrid esophagectomy	583 (96.4)	239 (80.5)
LTA/open	22 (3.6)	0 (0.0)
Transhiatal	0 (0.0)	58 (19.5)
Type reconstruction		
Gastric conduit	604 (99.8)	283 (95.3)
Colon interposition graft	1 (0.2)	14 (4.7)

*BMI* body mass index, *MIE* minimally invasive esophagectomy, *LTA* left thoracoabdominal

**Table 2:** Postoperative characteristics

	Intrathoracic	Cervical	*p* Value
Factor	(*n* = 605) *n* (%)	(*n* = 297) *n* (%)	
pT stage			
pT0	150 (26.0)	67 (24.5)	0.431 (FE)
pT1	102 (17.8)	58 (21.2)	
pT2	102 (17.8)	40 (14.6)	
pT3	209 (36.5)	106 (38.6)	
pT4	9 (1.5)	3 (1.2)	
pN stage			0.703
pN0	334 (58.2)	167 (60.5)	
pN1	130 (22.6)	57 (20.7)	
pN2	70 (12.2)	29 (10.5)	
pN3	40 (7.0)	23 (8.3)	
Complications			
Overall complications	337 (55.7)	210 (70.7)	<0.001
Pulmonary complication	153 (25.3)	108 (36.4)	<0.001
Cardiac complication	132 (21.8)	59 (19.9)	0.544
Postoperative anastomotic leakage	64 (10.6)	85 (28.6)	<0.001
Urinary complication	18 (3.0)	10 (3.4)	0.838
Chyle leakage	70 (11.6)	32 (10.8)	0.739
Recurrent nerve complication	8 (1.3)	22 (7.4)	<0.001
Surgical-site infection	36 (6.0)	44(14.8)	<0.001
Anastomotic stenosis	111 (18.4)	148 (49.8)	<0.001
Median time to anastomotic stenosis: days (range)	99.0 (35–1575)	77.5 (23–3864)	0.001
Median stenosis dilation: mm (range)	4 (1–184)	7 (1–28)	0.001

^a^Significant *p* value

## Results

Of the 902 patients, 605 underwent esophagectomy with an intrathoracic anastomosis. Of the 605 patients, 583 underwent an Ivor Lewis procedure, and 22 underwent esophagectomy via the left thoracoabdominal approach. Of the patients managed with a cervical anastomosis, 239 underwent a McKeown esophagectomy and 58 underwent a transhiatal esophagectomy. Minimally invasive esophagectomy was performed in 822 esophagectomies (91.1%), with the follow-up period ranging from 1 to 10 years.

### Benign Anastomotic Stenosis

Benign anastomotic stenosis was observed in 18.4% of the intrathoracic anastomosis group and in 49.8% of the cervical anastomosis group (*p* < 0.001). The stenosis rates for the different cervical techniques were as follows: end-to-side hand-sewn group (36.2%, 34/94), end-to-end one-layer hand-sewn group (58.8%, 60/102), end-to-end two-layer hand-sewn group (57.5%, 42/73), and end-to-side circular stapled group (42.9%, 12/28) (*p* = 0.006; Table 3).

**Table 3:** Cervical anastomotic technique

	End-to-side circular stapler (25 mm or unknown mm) (*n* = 28) *n* (%)	End-to-end 1 layer hand-sewn (*n* = 102) *n* (%)	End-to-end 2 layers hand-sewn (*n* = 73) *n* (%)	End-to-side hand-sewn (*n* = 94) *n* (%)	*p* Value
Postoperative complications	21 (75.0)	70 (68.6)	52 (71.2)	67 (71.3)	0.922
Anastomotic leakage	6 (21.4)	42 (41.2)	15 (20.5)	22 (23.4)	0.007^a^
Stenosis	12 (42.9)	60 (58.8)	42 (57.5)	34 (36.2)	0.006^a^

^a^Significant *p* value

The incidence of stenosis in the intrathoracic anastomosis group varied significantly among the different anastomotic techniques. Specifically, the 25 mm end-to-side circular stapler group exhibited a stenosis rate of 26.0%, whereas the 29 mm end-to-side circular stapler group had a stenosis rate of 13.5%. The stenosis rate for the patients treated with an unknown stapler size was 17.3%, in the end-to-side circular stapled group and 13.3% in the side-to-side linear stapled group (*p* = 0.001; Table 4). No significant difference was observed in complications and anastomotic leak rates.

**Table 4:** Intrathoracic anastomotic technique

	End-to-side circular stapler (25 mm) (*n* = 220) *n* (%)	End-to-side circular stapler (29 mm) (*n* = 296) *n* (%)	End-to-side circular stapler (unknown mm) (*n* = 75) *n* (%)	Side-to-side linear hand-sewn (*n* = 15) *n* (%)	*p* Value
Postoperative complications	122 (55.5)	161 (54.4)	47 (62.7)	8 (53.3)	0.428
Anastomotic leakage	26 (11.8)	27 (9.1)	10 (13.3)	2 (13.3)	0.441
Stenosis	57 (26.0)	40 (13.5)	13 (17.3)	2 (13.3)	0.001^a^

^a^Significant *p* value

The median time to stenosis was 99.0days for the intrathoracic and 77.5 days for the cervical cases (*p* = 0.001). The median number of dilations performed were four for intrathoracic and seven for cervical stenoses (*p* = 0.001; Table 2).

### Risk Factors for Stenosis

Risk factors for stenosis were analyzed separately for intrathoracic and cervical anastomosis. The univariable analysis is described in Tables S5, S6, S7, S8, S9, S10, S11, S12, S13, S14, S15, and S16.

*Intrathoracic Anastomosis.* After esophagectomy with intrathoracic anastomosis, multivariable analysis identified immunosuppressive medication as an independent risk factor (odds ratio [OR], 2.936; 95 % confidence interval [CI], 1.105–7.802; *p* = 0.031) and anastomotic leakage as a significant predictor of stenosis (OR, 2.034; 95% CI, 1.116–3.708; *p* = 0.020). Patients 70 years of age or older had a lower risk of stenosis (OR, 0.586; 95 % CI, 0.363–0.946; *p* = 0.029). A 29 m stapler reduced stenosis risk compared with a 25 mm stapler (OR, 0.494; 95% CI, 0.300–0.815; *p* = 0.006; Table S7).

In the intrathoracic anastomosis cohort without postoperative leakage, the patients 70 years of age or older had a lower stenosis risk (OR, 0.587; 95% CI, 0.357–0.966; *p* = 0.020). Cardiac complications also were associated with reduced stenosis risk (OR, 0.486; 95% CI, 0.239–0.991; *p* = 0.047), whereas chronic pulmonary disease (OR, 2.717; 95% CI, 1.293–5.707; *p* = 0.008) and immunosuppressive medication (OR, 3.068; 95% CI, 1.052–8.949; *p* = 0.040) were risk factors. A 29 mm stapler further reduced stenosis risk compared with a 25 mm stapler (OR, 0.486; 95% CI, 0.294–0.803; *p* = 0.005; Tables S11, S12, and S13).

*Cervical Anastomosis.* Anastomotic leakage was not significantly associated with stenosis in either the uni- or multivariable analysis. The risk of stenosis was independently reduced by pulmonary complications postoperatively, postoperative chyle leakage, and the use of an end-to-side hand-sewn technique (Table S9 and S10). In the cervical anastomosis cohort without anastomotic leakage, using an end-to-side hand-sewn technique significantly reduced stenosis occurrence (OR, 0.428; 95% CI, 0.207–0.885; *p* = 0.022; Table S16). Results were consistent when only transhiatal esophagectomy or McKeown procedures were included and colonic interpositions were excluded.

### Comment

This study investigated the risk factors for benign anastomotic stenosis after esophagectomy for cancer. The overall incidence of anastomotic stenosis in this series was 28.7% (18.4 % for intrathoracic and 49.8% for cervical stenosis; *p* < 0.001). Factors contributing to stenosis after esophageal resection are use of immunosuppressants and chronic pulmonary disease. Esophagectomy procedures involving intrathoracic anastomosis using a circular stapler with a larger diameter are associated with a decreased likelihood of stenosis occurrence, and anastomotic leakage is associated with an increased likelihood of stenosis occurrence. In cervical anastomosis, end-to-side hand-sewn technique reduced the risk for stenosis. Additionally, the treatment of a stenosis after esophagectomy with cervical anastomosis is more challenging, requiring a higher median number of dilations for effective treatment.

Despite substantial progress in pre-, peri-, and postoperative care, the overall incidence of anastomotic stenosis has seen only minimal improvement over time. In the current study, the incidence of anastomotic stenosis after esophagectomy with intrathoracic anastomosis was 18.4%, consistent with the reported range of 6–29 % in the literature.^21–24^ However, the rate of anastomotic stenosis for cervical anastomosis was higher (49.8%), surpassing the literature’s range of 0–40.^24–28^

The anastomosis method has long been central in assessing benign stenosis incidence. A major study on cervical anastomoses found no significant difference in stricture rates between hand-sewn end-to-end and end-to-side techniques, in contrast to our findings.^29^ In the current study, the 29 mm stapler in intrathoracic anastomosis showed a lower stenosis risk than the 25 mm stapler, aligning with studies indicating higher stenosis rates for smaller staplers.^30,31^ However, previous studies reported more stenosis with stapling than with hand-sewn methods.^15,16^ In the current study, no significant difference between linear and circular stapling techniques was observed, although few patients received linear stapling.

Chronic pulmonary disease, anastomotic leakage, and smaller stapler size were consistent risk factors, matching prior research.^9,10,21,23,32^ Contrary to previous findings, our study showed that an age of 70 years or older serves as an independent negative predictor.^27^ Currently, the literature on postoperative chyle leakage as a predictor for anastomotic stenosis is limited. However, we hypothesize that chyle leakage might indicate more extensive damage to thoracic lymphatic vessels, potentially aiding tissue healing and reducing fibrosis around the anastomotic site, although this complication is highly likely to be a confounder when considered as a protective factor. This study also found that immunosuppressive medication independently increases the risk of stenosis, likely due to its impact on healing at the anastomosis site, thereby contributing to the development of stenosis.

Studies have shown that anastomotic leakage significantly increases stenosis risk. ^10,33,34^ Compromised blood flow and oxygenation have an impact on tissue-healing after surgery, and leaked material may trigger inflammation, leading to scar tissue formation. This theory is supported by the success of oral steroids in managing benign esophageal strictures, but contrasts with our findings on preoperative immunosuppressant use as a risk factor.^35–37^

Indocyanine green enhancement timing at the planned anastomotic site is a promising tool for identifying patients at high risk for anastomotic strictures after esophagectomy with gastric conduit reconstruction. Studies link lower indocyanine green enhancement levels to higher risks of leakage and stenosis.^38^ Implementing intraoperative perfusion assessment could further personalize patient care, offering tailored follow-up guidelines for high-risk individuals. Furthermore, the use of endoscopic vacuum therapy shows promise in reducing stenosis after anastomotic leakage. Although the literature suggests that vacuum treatment may provide protective benefits compared with other anastomotic leakage treatment methods,^39^ our series did not have sufficient data to confirm this effect. Additionally, Jogiat et al.^40^ suggested that gastric ischemic pre-conditioning could reduce both anastomotic leaks and strictures, potentially lowering post-esophagectomy morbidity.

This study was constrained by its retrospective design and reliance on data from a single tertiary referral center, which made it challenging to collect all relevant data on stenosis treatment after esophagectomy and and posed a potential risk for some patients to be lost to follow-up evaluation. Additionally, notable diversity exists among surgical techniques over time, although this likely reflects the broader surgical landscape. However, this limited our ability to make definitive conclusions regarding the causal impact of various surgical techniques on outcomes.

## Conclusions

This report outlines the key risk factors associated with anastomotic stenosis after esophagectomy. The most important risk factor for stenosis in intrathoracic anastomoses is anastomotic leakage. The diameter of the circular stapler and the technique used in hand-sewn techniques are potentially modifiable aspects of surgical technique. Additionally, patients using immunosuppressants preoperatively and those with chronic pulmonary disease should be monitored for their increased risk of stenosis development. Future research should explore the biologic mechanisms of anastomotic healing and stenosis formation, as well as preventive strategies. Randomized multicenter studies are essential to the development of evidence-based guidelines on surgical techniques and intraoperative interventions to reduce stenosis risk.

## Supplementary Information

Below is the link to the electronic supplementary material.

Supplementary file1 (DOCX 31 KB)

Supplementary file2 (DOCX 50 KB)

Supplementary file3 (DOCX 1293 KB)
