# Supplementary material for: Risk Factors for Benign Anastomotic Stenosis After Esophagectomy for Cancer
Source: Ann Surg Oncol. 2025 May 6;32(8):5919–27. doi: 10.1245/s10434-025-17401-x (PMC12222431; doi:10.1245/s10434-025-17401-x)
Supplement: Supplementary file 1 — Supplementary file1 (DOCX 31 KB) [file 10434_2025_17401_MOESM1_ESM.docx]

**SUPPLEMENTARY MATERIAL**

**SURGERY TECHNIQUES**

**Transthoracic esophagectomy**

During the laparoscopic, robotic or open abdominal phase, an upper abdominal lymph node dissection (stations 14, 15, 16, 17, 18 and 19 according to TIGER-classification (1), Supplement 1) is performed, followed by mobilization of the stomach, and creation of a 3-4 cm wide gastric conduit and placement of a feeding jejunostomy.

In case of an intrathoracic anastomosis, the thoracic esophagus is mobilized, the thoracic duct resected and a mediastinal lymph node dissection is performed (station 8, 9, 11, 12, and 13 according to TIGER-classification (1)). This is followed by pulling up the gastric conduit, extraction of the specimen, and the creation of the anastomosis.

In case of a cervical anastomosis, the operation starts with the thoracic phase: either thoracoscopic, robotic or open mobilization of the esophagus is performed, including thoracic duct resection and mediastinal (stations 4, 5, 8, 9, 10, 11, 12 and 13), and on indication cervical lymph node dissection (TIGER station 2 (1)). Next, the abdominal phase is performed as described above, followed by a cervical incision, usually on the left side, unless there is an indication for the right side (e.g. location of lymph node metastases on the right recurrent laryngeal nerve). After the mobilization of the cervical esophagus with respect for the recurrent laryngeal nerves, the anastomosis is created.

**Transhiatal esophagectomy**

During transhiatal esophagectomy the abdominal phase is conducted as described above, followed by a transhiatal mediastinal dissection up to the level of the pulmonary veins/distal part of carina. If necessary, the hiatus is widened ventrally by transecting the phrenic vein. The anastomosis is created in the neck as described above.

**Colonic interposition**

In case of a colonic interposition, the right hemicolon, including the terminal ileum, is preferred, but if after clamping of the ileocolic (and if indicated right colic vessels), this does not remain vital, the left colon is used. A cervical (rarely intrathoracic) esophago-ileostomy, an intra-abdominal colo-jejunostomy, jejuno-jejunostomy and ileo-transversostomy are then created. The conduit is either placed prevertebrally, retrosternally or rarely subcutaneously.

**Anastomotic techniques**

Several anastomotic techniques and modifications were implemented and used during the study period. The timeline spans from the introduction of open McKeown and transhiatal esophagectomy, followed by the initiation of neoadjuvant therapy, to the adoption of minimally invasive esophagectomy (MIE) techniques (e.g McKeown, Ivor Lewis, and transhiatal), the beginning of flap and wrap procedures, and in 2019, the start of robot-assisted MIE, with the current techniques including end-to-side circular stapler for Ivor Lewis and end-to-end two-layer hand-sewn for McKeown (detailed info Figure 2.)

**Intrathoracic anastomosis**

Either a hand-sewn, circular stapled or linear stapled anastomosis is performed.

Hand-sewn anastomoses is performed in an end-to-end fashion with running sutures PDS 3.0.

Circular stapled anastomoses is performed in an end-to-side fashion with either a 25- or 29-mm Echelon (Power) circular stapler (J&J). The anastomosis is oversewn with two interrupted PDS 3.0 sutures (open), Vicryl 3.0 sutures (minimally invasive) or continuous sutures with V-loc (robotic).

Linear stapled anastomoses are made side-to-side fashioned with a blue cartridge of a (Powered) Echelon Flex 60 mm linear stapler (J&J), and the remaining defect is closed with a 3.0 V-loc.

Following a hand-sewn, linear or circular stapled anastomosis, a pleural flap fixation and an omental wrap-plasty is performed since 2014, as described in earlier studies from out institute (12). Our current standard anastomotic method is an end-to-side circular stapled anastomosis, except for one surgeon (FD), who performs a side-to-side linear (semi-mechanical) stapled anastomosis.

**Cervical anastomosis**

The end-to-end, one-layer anastomosis is sutured with continuous PDS 3.0. Circular stapled anastomoses is performed with an end-to-side or end-to-end fashion with a 25- or 29-mm Echelon (Power) circular stapler (J&J). Our current standard of all surgeons is the end-to-end, two-layer technique: it consists of the application of interrupted sutures from the serosa of the gastric conduit to the muscular layer of the esophagus utilizing 4.0 Vicryl. Additionally, mucosa-to-mucosa suturing is performed with continuous suturing using PDS 5.0. Following all cervical anastomosis from 2014 onwards an omental wrap-plasty were performed (2).

In the colonic interpositions the cervical anastomosis is manually constructed using an end-to-end two-layered technique Vicryl 4.0 interrupted for muscle layer and continuous PDS 5.0 for the mucosa (single layer PDS 3.0 before 2020). Subsequent continuity is established through a colo-jejunal anastomosis, incorporating a Roux-Y limb, and an ileo-colonic anastomosis. Closure of mesenteric defects and creation of a feeding jejunostomy concluded the procedure.

**REFERENCES**

1. Hagens ERC, van Berge Henegouwen MI, van Sandick JW, Cuesta MA, van der Peet DL, Heisterkamp J, et al. Distribution of lymph node metastases in esophageal carcinoma [TIGER study]: study protocol of a multinational observational study. BMC Cancer. 2019;19(1):662.

2. Slaman AE, Eshuis WJ, van Berge Henegouwen MI, Gisbertz SS. Improved anastomotic leakage rates after the "flap and wrap" reconstruction in Ivor Lewis esophagectomy for cancer. Dis Esophagus. 2022;36(1).
