# Supplementary material for: Risk Factors for Benign Anastomotic Stenosis After Esophagectomy for Cancer
Source: Ann Surg Oncol. 2025 May 6;32(8):5919–27. doi: 10.1245/s10434-025-17401-x (PMC12222431; doi:10.1245/s10434-025-17401-x)
Supplement: Supplementary file 2 — Supplementary file2 (DOCX 50 KB) [file 10434_2025_17401_MOESM2_ESM.docx]

**SUPPLEMENTARY MATERIAL**

**TABLES**

**Supplementary Table 5.** Preoperative factors for stenosis for an intrathoracic anastomosis

|  | **Univariable** | | | **Multivariable** | | |
| --- | --- | --- | --- | --- | --- | --- |
| **Variable** | **OR** | **95% CI** | **p-value** | **OR** | **95% CI** | **p-value** |
| Sex (male/female) | 0.855 | 0.492-1.487 | 0.579 | *-* | - | - |
| Age >= 70 | 0.583 | 0.362-0.939 | 0.027* | 0.586 | 0.363-0.946 | 0.029* |
| BMI >25 | 0.922 | 0.610-1.393 | 0.699 | - | - | - |
| BMI <20 | 0.880 | 0.400-1.936 | 0.750 | *-* | - | - |
| Hypertension | 0.740 | 0.370-1.481 | 0.395 | 0.242 | 0.032-1.856 | 0.175 |
| Chronic pulmonary disease | 2.047 | 1.031-4.067 | 0.041* | 1.807 | 0.892-3.660 | 0.095 |
| Diabetes mellitus | 0.751 | 0.382-1.477 | 0.406 | - | - | - |
| Atrial fibrillation | 0.215 | 0.029-1.619 | 0.136 | 0.242 | 0.032-1.856 | 0.172 |
| Myocardial infarction | 1.426 | 0.556-3.656 | 0.461 | - | - | - |
| Peripheral arterial disease | 0.600 | 0.163-2.205 | 0.442 | - | - | - |
| Kidney disease | 1.640 | 0.305-8.815 | 0.981 | - | - | - |
| Immunosuppressive medication | 2.949 | 4.117-7.778 | 0.029* | 2.936 | 1.105-7.802 | 0.031* |
| Histology |  |  |  |  |  |  |
| Adenocarcinoma | ref |  |  | *ref* |  |  |
| Squamous cell carcinoma | 2.075 | 1.173-3.672 | 0.012* | 1.773 | 0.927-3.394 | 0.084 |
| Other histology | 0.813 | 0.179-3.695 | 0.788 | 0.743 | 0.163-3.397 | 0.702 |
| Neo-adjuvant therapy |  |  |  |  |  |  |
| No neo-adjuvant | ref |  |  | *ref* |  |  |
| Chemotherapy | 0.459 | 0.155-1.360 | 0.160 | 0.506 | 0.165-1.548 | 0.233 |
| Chemoradiotherapy | 0.618 | 0.308-1.239 | 0.175 | *0.621* | 0.299-1.290 | 0.201 |
| Abbreviations: BMI= body mass index. * = significant p-value | | | | | | |

**Supplementary Table 6.** Postoperative factors for stenosis for an intrathoracic anastomosis

|  | **Univariate** | | | **Multivariate** | | |
| --- | --- | --- | --- | --- | --- | --- |
| **Variable** | **OR** | **95% CI** | **p-value** | **OR** | **95% CI** | **p-value** |
| Anastomotic leakage | 2.243 | 1.263-3.984 | 0.006* | 2.034 | 1.116-3.708 | 0.020* |
| Pulmonary complication | 0.388 | 0.882-2.183 | 0.156 | 1.134 | 0.696-1.846 | 0.614 |
| Cardiac complication | 0.803 | 0.477-1.351 | 0.408 | - | - | - |
| Postoperative chyle leakage | 0.909 | 0.470-1.757 | 0.777 | - | - | - |
| Postoperative SSI | 2.059 | 0.981-4.322 | 0.056 | 1.612 | 0.738-3.520 | 0.231 |
| ICU admission (yes/no) | 1.224 | 0.788-1.900 | 0.368 | - | - | - |
| Abbreviations: SSI= surgical site infection; * = significant p-value | | | | | | |

**Supplementary Table 7.** Surgical modifiable factors for an intrathoracic anastomosis

|  | **Univariable** | | | **Multivariable** | | |
| --- | --- | --- | --- | --- | --- | --- |
| **Variable** | **OR** | **95% CI** | **p-value** | **OR** | **95% CI** | **p-value** |
| Anastomotic configurations |  |  |  |  |  |  |
| End to side circular stapler | ref | - | - | - | - | - |
| Side to side hand-sewn | 0.676 | 0.150-3.040 | 0.610 | *-* | - | - |
| Size stapler |  |  |  |  |  |  |
| Stapler size 25mm | ref |  |  | - | - | - |
| Stapler size 29mm | 0.444 | 0.283-0.696 | <0.001* | - | - | - |
| Stapler size unknown | 0.596 | 0.305-1.164 | 0.130 | - | - | - |
| Reconstruction route |  |  |  |  |  |  |
| Prevertebral | ref |  |  | - | - | - |
| Retrosternal | 0.964 | 0.205-4.528 | 0.963 | - | - | - |
| * = significant p-value | | | | | | |

**Supplementary Table 8.** Preoperative factors for stenosis for a cervical anastomosis

|  | **Univariable** | | | **Multivariable** | | |
| --- | --- | --- | --- | --- | --- | --- |
| **Variable** | **OR** | **95% CI** | **p-value** | **OR** | **95% CI** | **p-value** |
| Sex (male/female) | 1.328 | 0.818-2.154 | 0.251 | - | - | - |
| Age>70 | 0.656 | 0.395-1.090 | 0.104 | 0.639 | 0.383-1.065 | 0.086 |
| BMI>25 | 0.957 | 0.604-1.517 | 0.853 | - | - | - |
| BMI<20 | 1.151 | 0.563-2.354 | 0.700 | - | - | - |
| Hypertension | 1.304 | 0.433-3.925 | 0.637 | - | - | - |
| Chronic pulmonary disease | 0.699 | 0.300-1.627 | 0.406 | - | - | - |
| Diabetes mellitus | 1.507 | 0.730-3.109 | 0.267 | - | - | - |
| Myocardial infarction | 1.021 | 0.062-16.897 | 0.988 | - | - | - |
| Immunosuppressive medication | 1.521 | 0.250-9235 | 0.649 | - | - | - |
| Histology |  |  |  |  |  |  |
| Adenocarcinoma | ref |  |  |  |  |  |
| Squamous cell carcinoma | 1.412 | 0.866-2.304 | 0.167 | 1.376 | 0.836-2.266 | 0.210 |
| Other histology | 1.397 | 0.573-3.408 | 0.462 | 1.006 | 0.384-2.638 | 0.990 |
| Neo-adjuvant therapy |  |  |  |  |  |  |
| No neo-adjuvant | ref |  |  |  |  |  |
| Chemotherapy | 0.141 | 0.015-1.303 | 0.084 | 0.131 | 0.014-1.218 | 0.074 |
| Chemoradiotherapy | 0.676 | 0.357-1.279 | 0.228 | 0.667 | 0.351-1.267 | 0.216 |
| Abbreviations: BMI= body mass index | | | | | | |

**Supplementary Table 9.** Postoperative factors for stenosis for a cervical anastomosis

|  | **Univariable** | | | **Multivariable** | | |
| --- | --- | --- | --- | --- | --- | --- |
| **Variable** | **OR** | **95% CI** | **p-value** | **OR** | **95% CI** | **p-value** |
| Anastomotic leakage | 0.977 | 0.590-1.616 | 0.927 | - | - | - |
| Pulmonary complication | 0.529 | 0.327-0.856 | 0.009* | 0.561 | 0.345-0.912 | 0.020* |
| Cardiac complication | 0.888 | 0.502-1.572 | 0.684 | - | - | - |
| Postoperative chyle leakage | 0.355 | 0.158-0.795 | 0.012* | 0.388 | 0.171-0.878 | 0.023* |
| Postoperative SSI | 0.524 | 0.270-1.015 | 0.056 | 0.699 | 0.347-1.409 | 0.317 |
| ICU admission (yes/no) | 0.704 | 0.441-1.124 | 0.141 | 0.739 | 0.456-1.199 | 0.221 |
| Abbreviations: SSI= surgical site infection; * = significant p-value | | | | | | |

**Supplementary Table 10.** Surgical modifiable factors for stenosis for a cervical anastomosis

|  | **Univariable** | | | **Multivariable** | | |
| --- | --- | --- | --- | --- | --- | --- |
| **Variable** | **OR** | **95% CI** | **p-value** | **OR** | **95% CI** | **p-value** |
| Anastomotic technique |  |  |  |  |  |  |
| End-to-end 2 layers | ref |  |  |  |  |  |
| End-to-end 1 layer | 1.054 | 0.573-1.939 | 0.783 | 1.073 | 0.564-2.041 | 0.830 |
| End-to-side hand-sewn | 0.418 | 0.224-0.783 | 0.006* | 0.454 | 0.234-0.879 | 0.019* |
| End-to-side circular stapler | 0.554 | 0.229-1.336 | 0.188 | 0.534 | 0.215-1.330 | 0.178 |
| Reconstruction |  |  |  |  |  |  |
| Gastric tube | ref |  |  |  |  |  |
| Colon interposition | 0.690 | 0.255-1.864 | 0.464 | - | - | - |
| Reconstruction route |  |  |  |  |  |  |
| Prevertebral | ref |  |  |  |  |  |
| Retrosternal | 1.007 | 0.368-2.761 | 0.989 | - | - | - |
| * = significant p-value | | | | | | |

**Supplementary Table 11.** Pre-operative factors for stenosis for an intrathoracic anastomosis without postoperative anastomotic leakage

|  | **Univariable** | | | **Multivariable** | | |
| --- | --- | --- | --- | --- | --- | --- |
| **Variable** | **OR** | **95% CI** | **p-value** | **OR** | **95% CI** | **p-value** |
| Sex (male/female) | 0.779 | 0.420-1.442 | 0.426 | - | - | - |
| Age >= 70 | 0.531 | 0.312-0.906 | 0.020* | 0.587 | 0.357-0.966 | 0.020* |
| BMI <20 | 0.688 | 0.262-1.806 | 0.447 | - | - | - |
| BMI >25 | 0.844 | 0.538-1.325 | 0.462 | - | - | - |
| Hypertension | 0.744 | 0.377-1.469 | 0.395 | - | - | - |
| Chronic pulmonary disease | 2.576 | 1.243-5.341 | 0.011* | 2.717 | 1.293-5.707 | 0.008* |
| Diabetes mellitus | 0.597 | 0.263-1.357 | 0.218 | - | - | - |
| Atrial fibrillation | 0.266 | 0.035-2.019 | 0.200 | - | - | - |
| Myocardial infarction | 1.573 | 0.561-4.409 | 0.389 | - | - | - |
| Peripheral arterial disease | 0.608 | 0.137-2.692 | 0.512 | - | - | - |
| Kidney disease | 1.659 | 0.330-8.354 | 0.539 | - | - | - |
| Immunosuppressive medication | 3.451 | 1.197-9.949 | 0.022* | 3.068 | 1.052-8.949 | 0.040* |
| Histology |  |  |  |  |  |  |
| Adenocarcinoma | ref |  |  |  |  |  |
| Squamous cell carcinoma | 2.232 | 1.204-4.136 | 0.011* | 1.736 | 0.890-3.385 | 0.106 |
| Other | 1.103 | 0.237-5.138 | 0.901 | 0.534 | 0.066-4.346 | 0.558 |
| Neo-adjuvant therapy |  |  |  |  |  |  |
| No neo-adjuvant | ref |  |  |  |  |  |
| Chemotherapy | 0.627 | 0.201-1.962 | 0.423 | - | - | - |
| Chemoradiotherapy | 0.696 | 0.319-1.519 | 0.363 | - | - | - |
| Abbreviations: BMI= body mass index. SSI= surgical site infection; * = significant p-value | | | | | | |

**Supplementary Table 12.** Postoperative risk factors for stenosis for an intrathoracic anastomosis without postoperative anastomotic leakage

|  | **Univariable** | | | **Multivariable** | | |
| --- | --- | --- | --- | --- | --- | --- |
| **Variable** | **OR** | **95% CI** | **p-value** | **OR** | **95% CI** | **p-value** |
| Pulmonary complication | 0.970 | 0.558-1688 | 0.915 | - | - | - |
| Cardiac complication | 0.486 | 0.242-0.974 | 0.042* | 0.486 | 0.239-0.991 | 0.047* |
| Postoperative chyle leakage | 0.876 | 0.414-1.851 | 0.728 | - | - | - |
| Postoperative SSI | 1.573 | 0.561-4.409 | 0.389 | - | - | - |
| ICU admission (yes/no) | 0.832 | 0.489-1.414 | 0.497 | - | - | - |
| Abbreviations: SSI= surgical site infection; * = significant p-value | | | | | | |

**Supplementary Table 13.** Surgical modifiable factors for stenosis for an intrathoracic anastomosis without postoperative anastomotic leakage

|  | **Univariable** | | | **Multivariable** | | |
| --- | --- | --- | --- | --- | --- | --- |
| **Variable** | **OR** | **95% CI** | **p-value** | **OR** | **95% CI** | **p-value** |
| Anastomotic configurations |  |  |  |  |  |  |
| End to side circular stapler | ref |  |  |  |  |  |
| Side to side hand-sewn | 0.893 | 0.195-4.097 | 0.884 | - | - | - |
| Size stapler |  |  |  |  |  |  |
| Stapler size 25mm | ref |  |  |  |  |  |
| Stapler size 29mm | 0.476 | 0.291-0.777 | 0.003* | 0.486 | 0.294-0.803 | 0.005* |
| Stapler size unknown | 0.670 | 0.323-1.389 | 0.282 | 0.646 | 0.299-1.396 | 0.267 |
| Reconstruction route |  |  |  |  |  |  |
| Prevertebral | ref |  |  |  |  |  |
| Retrosternal | 1.383 | 0.282-6.69 | 0.689 | - | - | - |
| * = significant p-value | | | | | | |

**Supplementary Table 14.** Preoperative factors for stenosis for a cervical anastomosis without postoperative anastomotic leakage

|  | **Univariable** | | | **Multivariable** | | |
| --- | --- | --- | --- | --- | --- | --- |
| **Variable** | **OR** | **95% CI** | **p-value** | **OR** | **95% CI** | **p-value** |
| Sex (male/female) | 1.229 | 0.699-2.161 | 0.473 | - | - | - |
| Age>70 | 0.696 | 0.387-1.259 | 0.232 | - | - | - |
| BMI <20 | 1.496 | 0.676-3.309 | 0.320 | - | - | - |
| BMI >25 | 0.855 | 0.494-1.480 | 0.576 | - | - | - |
| Hypertension | 1.178 | 0.382-3.631 | 0.775 | - | - | - |
| Chronic pulmonary disease | 0.647 | 0.222-1.885 | 0.425 | - | - | - |
| Diabetes mellitus | 1.418 | 0.547-3.680 | 0.472 | - | - | - |
| ICU admission (yes/no) |  |  |  |  |  |  |
| Histology |  |  |  |  |  |  |
| Adenocarcinoma | ref |  |  |  |  |  |
| Squamous cell carcinoma | 1.216 | 0.675-2.189 | 0.515 | - | - | - |
| Other histology | 1.235 | 0.423-3.606 | 0.699 | - | - | - |
| Neo-adjuvant therapy |  |  |  |  |  |  |
| No neo-adjuvant | ref |  |  |  |  |  |
| Chemotherapy | 1.000 |  |  |  |  |  |
| Chemoradiotherapy | 0.741 | 0.356-1.543 | 0.741 | - | - | - |
| Abbreviations: BMI= body mass index. SSI= surgical site infection; * = significant p-value | | | | | | |

**Supplementary Table 15.** Postoperative risk factors for stenosis for a cervical anastomosis without postoperative anastomotic leakage

|  | **Univariable** | | | **Multivariable** | | |
| --- | --- | --- | --- | --- | --- | --- |
| **Variable** | **OR** | **95% CI** | **p-value** | **OR** | **95% CI** | **p-value** |
| Pulmonary complication | 0.654 | 0.57-1.198 | 0.169 | 0.686 | 0.372-1.266 | 0.228 |
| Cardiac complication | 0.888 | 0.452-1.746 | 0.730 | - | - | - |
| Postoperative chyle leakage | 0.495 | 0.200-1.223 | 0.128 | 0.526 | 0.211-1.310 | 0.168 |
| ICU admission (yes/no) | 0.891 | 0.518-1.534 | 0.678 | - | - | - |
| Abbreviations: SSI= surgical site infection | | | | | | |

**Supplementary Table 16.** Surgical modifiable risk factors for stenosis for a cervical anastomosis without postoperative anastomotic leakage

|  | **Univariable** | | | **Multivariable** | | |
| --- | --- | --- | --- | --- | --- | --- |
| **Variable** | **OR** | **95% CI** | **p-value** | **OR** | **95% CI** | **p-value** |
| Anastomotic technique |  |  |  |  |  |  |
| End-to-end 2 layers | ref |  |  | ref |  |  |
| End-to-end 1 layer | 1.219 | 0.584-2.543 | 0.598 | 1.322 | 0.617-2.832 | 0.472 |
| End-to-side hand-sewn | 0.455 | 0.224-0.920 | 0.028* | 0.428 | 0.207-0.885 | 0.022* |
| End-to-side circular stapler | 0.524 | 0.194-1.420 | 0.204 | 0.503 | 0.184-1.376 | 0.181 |
| Reconstruction |  |  |  |  |  |  |
| Gastric tube | ref |  |  |  |  |  |
| Colon interposition | 0.357 | 0.92-1.384 | 0.136 | 0.531 | 0.113-2.497 | 0.423 |
| Reconstruction route |  |  |  |  |  |  |
| Prevertebral | ref |  |  |  |  |  |
| Retrosternal | 0.416 | 0.104-1.654 | 0.213 | 0.379 | 0.085-1.701 | 0.205 |
| Abbreviations: * = significant p-value | | | | | | |
