# Supplementary figures and images for: Risk Factors for Benign Anastomotic Stenosis After Esophagectomy for Cancer
Source: Ann Surg Oncol. 2025 May 6;32(8):5919–27. doi: 10.1245/s10434-025-17401-x (PMC12222431; doi:10.1245/s10434-025-17401-x)

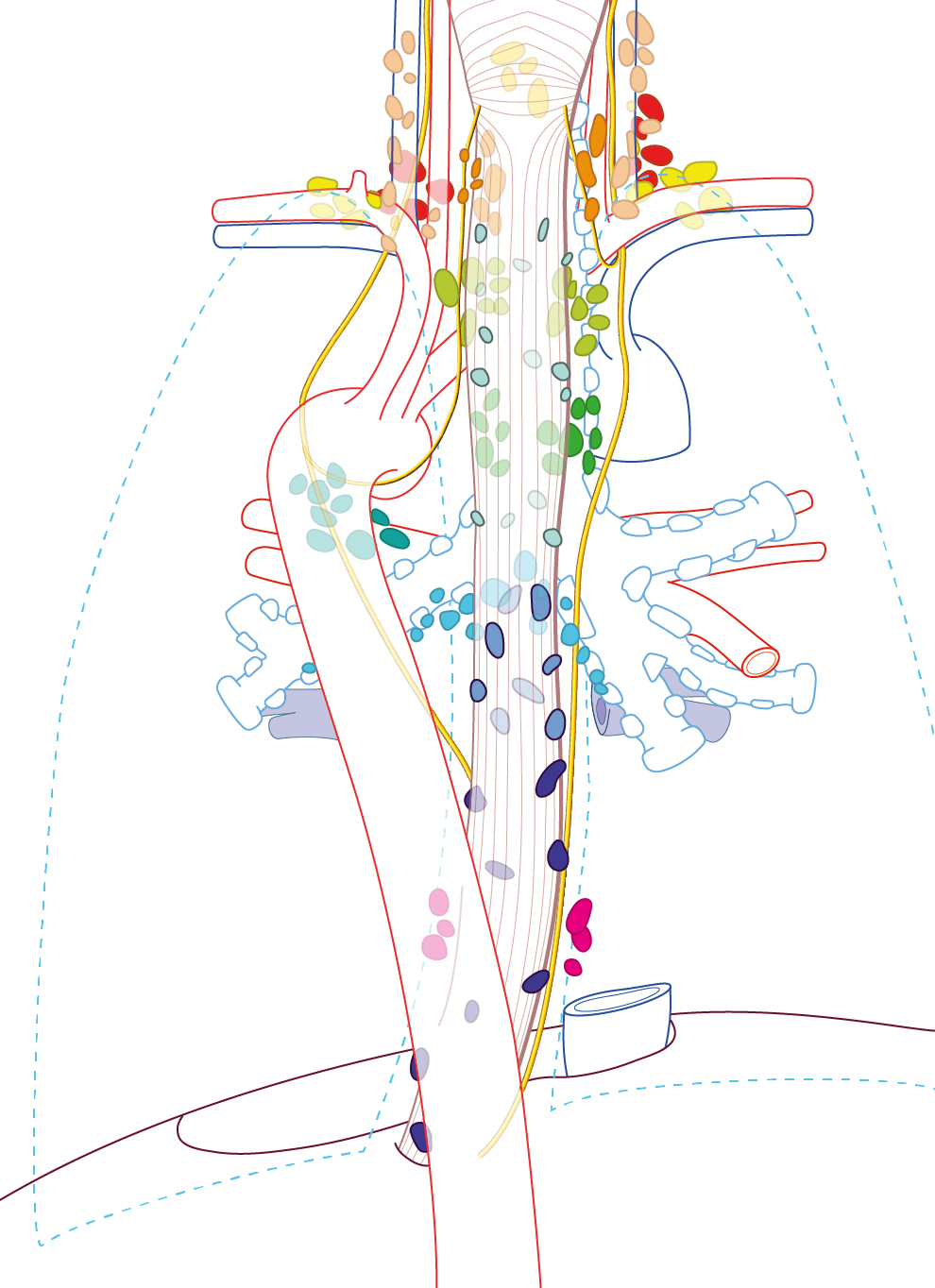

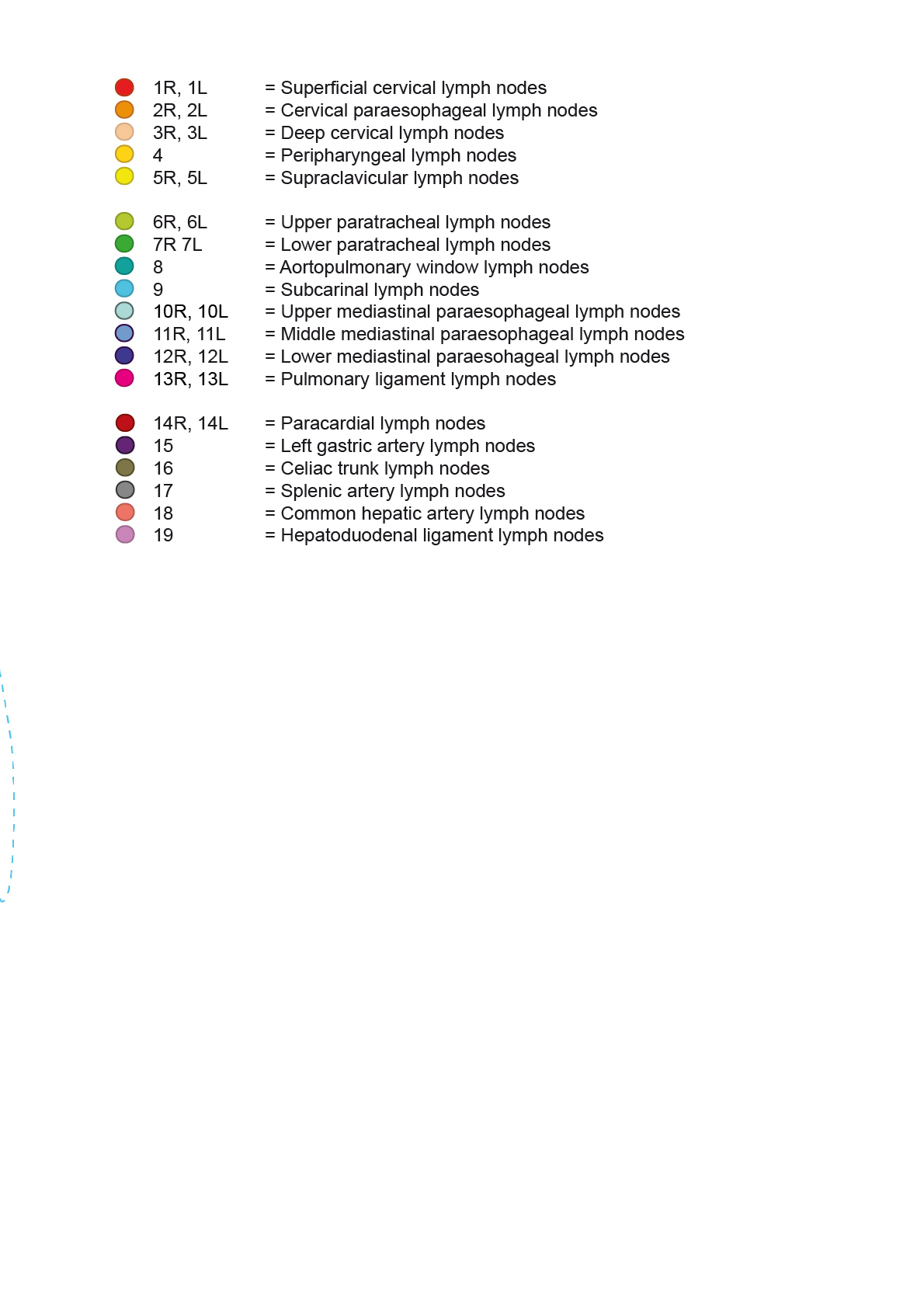

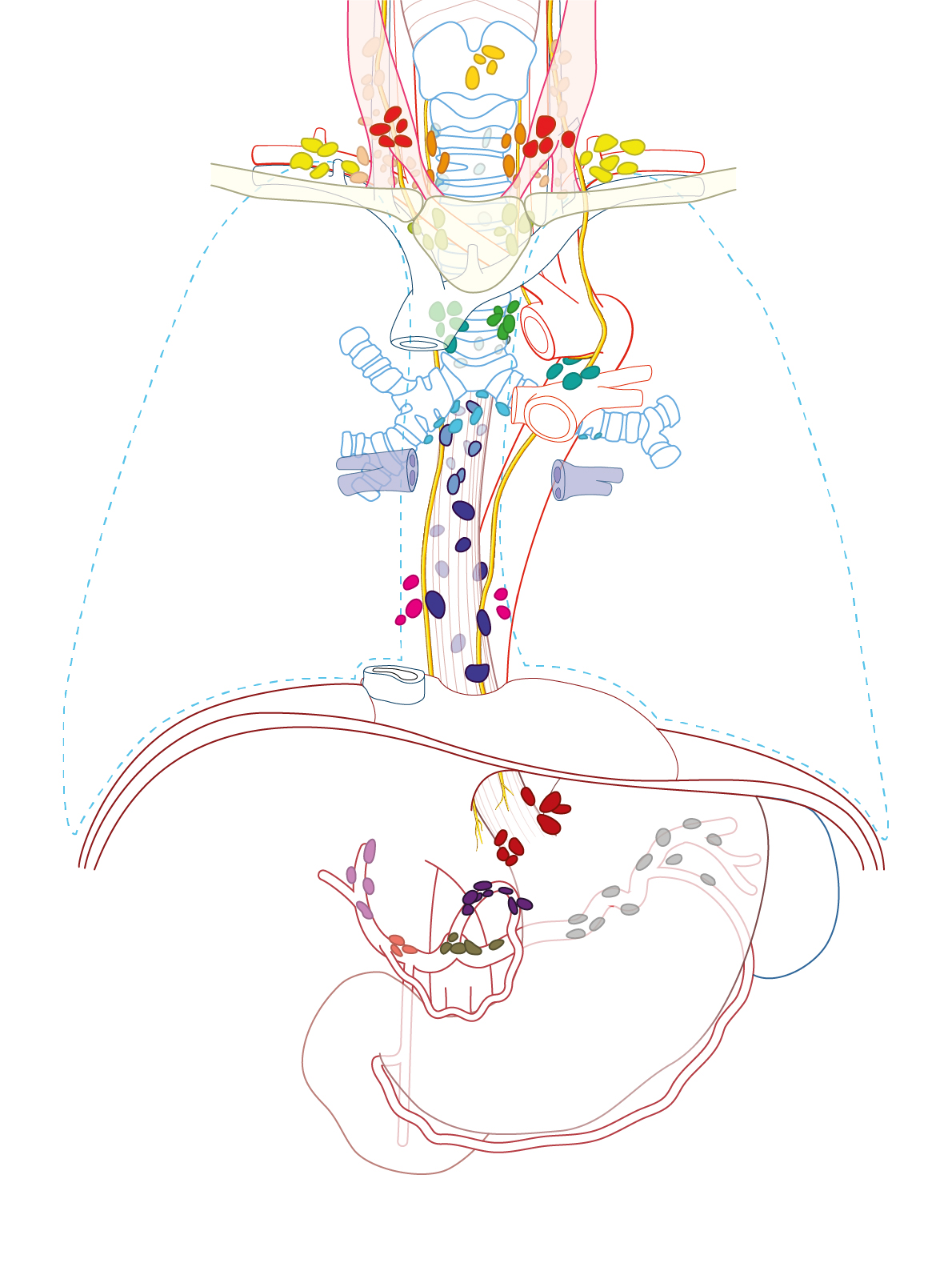

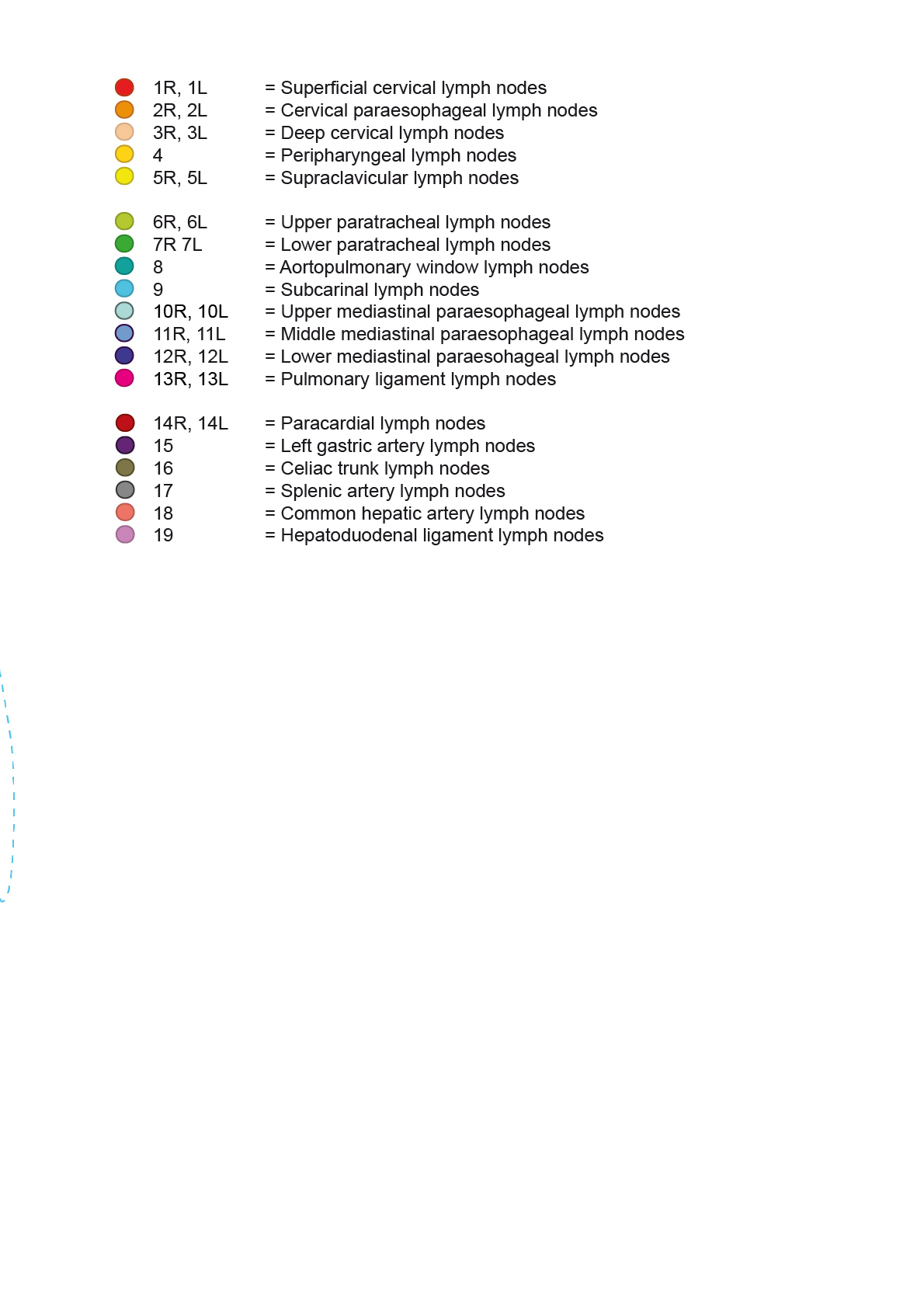

Supplement: Supplementary file 3 — Supplementary file3 (DOCX 1293 KB) [file 10434_2025_17401_MOESM3_ESM.docx]
